# Duloxetine Protects against Oxaliplatin-Induced Neuropathic Pain and Spinal Neuron Hyperexcitability in Rodents

**DOI:** 10.3390/ijms18122626

**Published:** 2017-12-05

**Authors:** Woojin Kim, Yeongu Chung, Seunghwan Choi, Byung-Il Min, Sun Kwang Kim

**Affiliations:** 1 Department of Physiology, College of Korean Medicine, Kyung Hee University, Seoul 02447, Korea; wjkim@khu.ac.kr; 2 Department of East-West Medicine, Graduate School, Kyung Hee University, Seoul 02447, Korea; yeongu.chung@gmail.com (Y.C.); choiret@gmail.com (S.C.); mbi@khu.ac.kr (B.-I.M.); 3 Department of Neurosurgery, College of Medicine, Kyung Hee University, Kyung Hee University Hospital, Seoul 02447, Korea; 4 Yeongju Municipal Hospital, Yeongju-si 36051, Korea

**Keywords:** chemotherapy-induced peripheral neuropathy, duloxetine, noradrenergic receptor, oxaliplatin, wide dynamic range (WDR) cell

## Abstract

Oxaliplatin is a widely used chemotherapy agent, but induces serious peripheral neuropathy. Duloxetine is a dual reuptake inhibitor of serotonin and norepinephrine, and is shown to be effective against pain. However, whether and how duloxetine can attenuate oxaliplatin-induced allodynia in rodents is not clearly understood. A single injection of oxaliplatin (6 mg/kg, intraperitoneal; i.p.) induced a cold and mechanical allodynia, which was assessed by acetone and von Frey filament tests, respectively. When significant allodynic signs were observed, three different doses of duloxetine (10, 30, and 60 mg/kg, i.p.) were injected. Administration of 30 and 60 mg/kg of duloxetine significantly reduced the allodynia, whereas 10 mg/kg did not. By using an in vivo extracellular recording method, we further confirmed that 30 mg/kg of duloxetine could significantly inhibit the hyperexcitability of spinal wide dynamic range (WDR) cells. The anti-allodynic effect of duloxetine was completely blocked by an intrathecal injection of phentolamine (non-selective α-adrenergic receptor antagonist, 20 μg), or prazosin (α_1_-adrenergic receptor antagonists, 10 μg); however, idazoxan (α_2_-adrenergic receptor antagonist, 10 μg) did not block it. In conclusion, we suggest that duloxetine may have an effective protective action against oxaliplatin-induced neuropathic pain and spinal hyperexcitability, which is mediated by spinal α_1_-adrenergic receptors.

## 1. Introduction

Oxaliplatin is a third-generation platinum-based chemotherapy agent widely used for advanced metastatic colorectal cancer [1,2], ovarian, breast, and lung cancer [3]. However, while it is efficacious against tumors, it induces severe acute peripheral neuropathy in about 90% of patients without causing any motor dysfunction even after a single infusion [4]. Thus, this acute peripheral neuropathy is the only major dose-limiting toxicity associated with oxaliplatin, which is characterized by peripheral dysesthesia and paresthesia of the hands and feet [4,5]. Even in rodents, a single intraperitoneal administration of oxaliplatin (6 mg/kg) can reproduce these neurotoxic profiles [6,7]. To attenuate this side effect, various drugs such as anti-convulsant agents and tricyclic antidepressants are used. However, these drugs also cause side effects, which prevent their wide use [8,9].

Duloxetine, a serotonin and noradrenaline re-uptake inhibitor, is a well-known antidepressant drug. However, it is also considered to be effective against various kinds of pain, such as fibromyalgia pain [10], osteoarthritis knee pain [11], and chronic lower back pain [12]. Furthermore, the U.S. Food and Drug Administration (FDA) has also approved the use of duloxetine for diabetic peripheral neuropathic pain [13]. Also, in a randomized, double-blind, and placebo-controlled crossover clinical trial, patients treated with duloxetine resulted in a greater reduction of chemotherapy-induced peripheral neuropathy (CIPN), compared to the placebo-treated group [14]. Regarding oxaliplatin-induced neuropathic pain, the American Society of Clinical Oncology has suggested that duloxetine may be beneficial for its treatment and prevention, based on several randomized clinical trials [15]. However, the site and mechanism of its action is not fully understood.

Previously, it was reported that neuropathic pain could be attenuated by the activation of spinal adrenergic receptors [16]. In accordance with this report, our study conducted on oxaliplatin-induced neuropathic pain rats showed that α_1_-, α_2_-adrenergic receptor agonist injected intrathecally could decrease the cold and mechanical allodynia induced by a single oxaliplatin injection. Furthermore, using in vivo extracellular recording, we also showed that α_1_-, α_2_-adrenergic receptor agonists could decrease the hyperexcitability of spinal wide dynamic range (WDR) cells evoked by oxaliplatin injection [17]. As a serotonin and noradrenaline re-uptake inhibitor, duloxetine is also known to act on adrenergic receptors. However, whether duloxetine acts on noradrenergic receptor and its subtypes present in the spinal cord has not yet been clearly investigated.

Thus, in this study, our first aim is to observe the time course of the analgesic effect of different doses of duloxetine on oxaliplatin-induced neuropathic pain. Secondly, by using in vivo extracellular recording, we aim to see whether duloxetine can decrease the hyperexcitability of WDR cells induced by oxaliplatin injection. Finally, our last aim is to clarify the role of spinal α-noradrenergic receptors in the analgesic effect of duloxetine.

## 2. Results

### 2.1. Effects of Different Doses of Duloxetine and Its Time Course on Oxaliplatin-Induced Cold and Mechanical Allodynia in Mice

**Figure 1:**
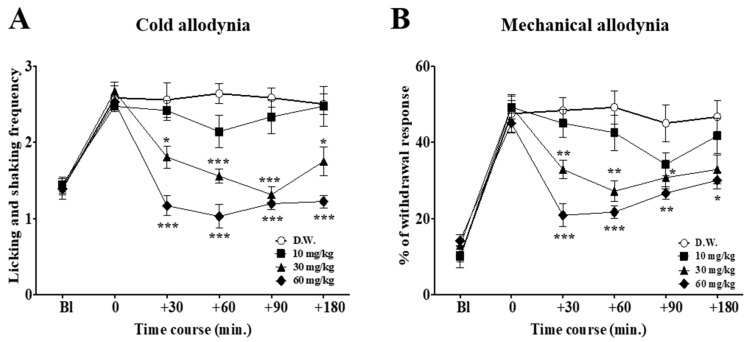
Time course of the effect of different doses of duloxetine on oxaliplatin-induced cold (**A**) and mechanical (**B**) allodynia in mice. Distilled water (D.W.) and three different doses of duloxetine (10, 30, and 60 mg/kg) were administered intraperitoneally (*n* = 7/group). D.W. was injected to control group mice. On the timeline, Bl refers to the assessment made before the injection of oxaliplatin, and 0 refers to the assessment made three days after the oxaliplatin injection, just prior to the administration of duloxetine. Bl: baseline. Data is presented as the standard error of the mean ± (S.E.M.); * *p* < 0.05, ** *p* < 0.01, *** *p* < 0.001 vs. D.W.; by Bonferroni post-test after one-way ANOVA.

On day 3, when the cold and mechanical allodynia signs were induced by a single administration of oxaliplatin (6 mg/kg, intraperitoneal; i.p.) [18], behavioral tests to assess the effect of different doses of duloxetine (10, 30, and 60 mg/kg) were conducted (Figure 1). Distilled water (D.W.) was used as a control. For cold allodynia assessment, the frequency of licking and shaking of the affected paw was counted during 30 s and averaged after the acetone had been sprayed. For mechanical allodynia measurement, a von Frey filament with a bending force of 0.4 g was applied to the mid-plantar skin of each hind paw 10 times, and the number of withdrawal responses to each application from both hind paws were counted and then expressed as an overall percentage response. Intraperitoneal administration of duloxetine showed dose-dependent effects. Medium and high doses (30 and 60 mg/kg, respectively) of duloxetine markedly reduced both the cold and mechanical allodynia. However, 10 mg/kg doses did not significantly reduce the mechanical and cold allodynia elicited by oxaliplatin injection. Thus, 30 mg/kg of duloxetine was shown to be the optimal dose in this experiment, as 10 mg/kg showed weak analgesic effect, and despite its strong analgesic effect somnolence, could be observed in some of the mice injected with 60 mg/kg.

### 2.2. Effects of Duloxetine on Increased Neuronal Response to Mechanical and Cold Stimulations Induced by Oxaliplatin Injection in the Spinal Dorsal Horn

**Figure 2:**
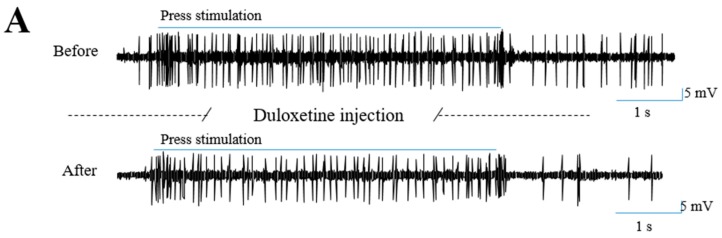

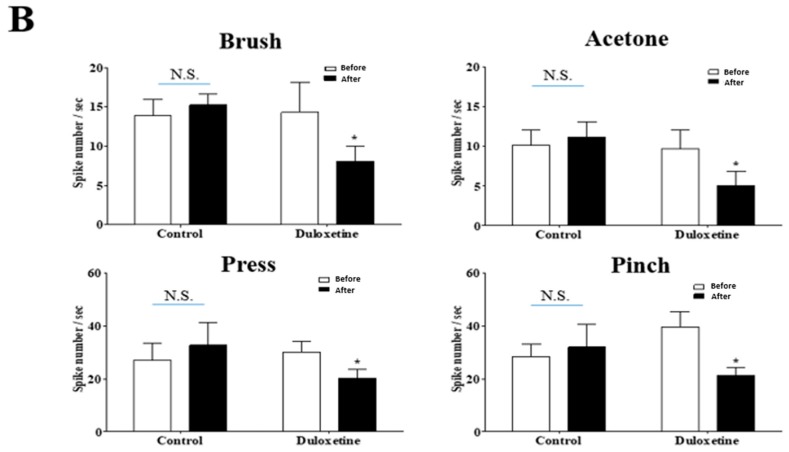
Duloxetine decrease the hyperexcitability of spinal wide dynamic range (WDR) cells induced by oxaliplatin. Representative extracellular recording raw traces of WDR neuron responses to press stimulation before and one hour after the intraperitoneal injection of 30 mg/kg of duloxetine (**A**). Frequency of neuronal activity to brush, press, pinch and cold stimulations were measured before and one hour after the administration of duloxetine (30 mg/kg, i.p., *n* = 6–7) (**B**). The same volume of D.W. was injected to the control group (*n* = 5–7) (**B**). N.S. refers to non-significant. Data is presented as mean ± S.E.M.; * p < 0.05 vs. Before; by paired *t*-test.

In our previous study, using in vivo extracellular recording methods, we reported that oxaliplatin significantly increased the firing frequency of WDR neurons in response to cold and mechanical stimulation in the spinal cord [17]. In this study, using the same extracellular recording method, we observed whether 30 mg/kg of duloxetine (i.p.) could decrease this increased neuronal activity in WDR neurons (Figure 2). Representative extracellular recording raw trace of WDR neurons to press (Figure 2A) stimulations demonstrated that the neuronal firing rate decreased one hour after duloxetine administration. In addition, the number of spike responses of the WDR neurons to mechanical (brush, press, and pinch) and cold (acetone drop) stimulation were significantly decreased after the injection of duloxetine compared to the responses shown before the injection (Figure 2B). However, the control group (D.W., i.p.) showed no significant change in WDR neuronal responses. These results show that 30 mg/kg of duloxetine treatment significantly reduced the augmented frequency of the WDR neurons in response to cold and mechanical stimulation elicited by oxaliplatin injection.

### 2.3. Effect of α-Adrenergic Receptor Antagonists on the Analgesic Effect of Duloxetine on Cold and Mechanical Allodynia in Mice

**Figure 3:**
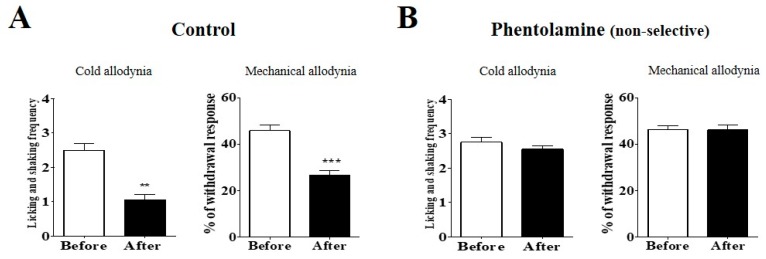

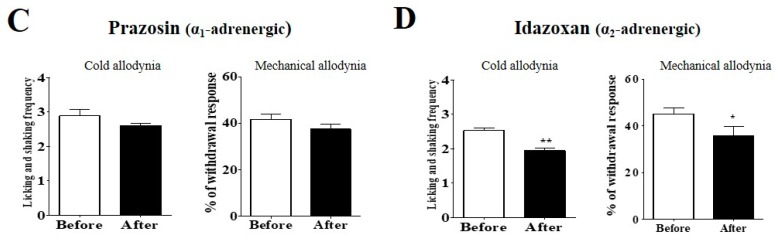
Effect of α-adrenergic receptor antagonists on the analgesic effect of duloxetine on cold and mechanical allodynia in mice. The behavioral tests for cold and mechanical allodynia measurements were performed 20 min prior to the pre-treatment of antagonist (Before) and 60 min after the intraperitoneal injection of 30 mg/kg of duloxetine (After). Phentolamine (non-selective α-adrenergic receptor antagonist, 20 μg, *n* = 7), prazosin (α_1_-adrenergic receptor antagonist, 10 μg, *n* = 6), and idazoxan (α_2_-adrenergic receptors, 10 μg, *n* = 6) were administered intrathecally (**B**–**D**, respectively). D.W. (*n* = 6) was injected to the control group (**A**). Data is presented as the mean ± S.E.M.; * *p* < 0.05, ** *p* < 0.01, *** *p* < 0.001 vs. Before; by paired *t*-test.

To clarify the analgesic mechanism of duloxetine at the spinal level, phentolamine (non-selective α-adrenergic receptor antagonist), prazosin (α_1_-adrenergic receptor antagonist), or idazoxan (α_2_-adrenergic receptor antagonist) was injected intrathecally 20 min before the injection of duloxetine (Figure 3). D.W. (Figure 3A), which was used for the control, did not block the anti-allodynic effect of the duloxetine, whereas phentolamine (Figure 3B) completely blocked the analgesic effect of duloxetine on both the cold and mechanical allodynia. To further clarify which noradrenergic receptor subtypes mediate the analgesic mechanism of duloxetine at the spinal level, α_1_- and α_2_-adrenergic antagonists were used. Prazosin (Figure 3C) completely blocked the anti-allodynic effect of the duloxetine, whereas idazoxan (Figure 3D) did not block the analgesic effect. These results indicate that spinal α_1_-adrenergic receptors but not α_2_-adrenergic receptors may be involved in the suppressive effect of duloxetine on oxaliplatin-induced cold and mechanical allodynia in mice.

## 3. Discussion

Oxaliplatin is a third-generation platinum-based chemotherapy drug that has gained importance in the treatment of advanced metastatic colorectal carcinoma [19]. However, it also induces a severe acute peripheral neuropathy [4], which is one of the major problems to successful chemotherapy treatment as it restricts both individual and cumulative dosages. Nevertheless, as an optimal substitute has not yet been found, oxaliplatin is commonly used and many patients still suffer from the development of peripheral neuropathy [4,20,21], which is characterized by a specific somatosensory profile like cold and mechanical allodynia. In humans, oxaliplatin is treated 85 mg/m^2^ every two weeks or 130 mg/m^2^ every three weeks, which corresponds approximately to the 6 mg/kg dose used in our experiments. Moreover, this dose of oxaliplatin was also reported to closely mimic the effects observed in humans even after a single infusion, especially onset and highly intense sensory disturbances, hypersensitivity to cold and mechanical allodynia, and hyperalgesia signs [7,22]. It is therefore important to find a drug that could have a protective effect against this neuropathic pain. However, first-choice drugs, such as gabapentin, anti-convulsant agents, and tricyclic antidepressants, have been reported to be unsatisfactory to CIPN as they also have side effects, which limit their use [23].

Duloxetine has been previously reported to be efficacious for the treatment of various pains [12]. Furthermore, the U.S. Food and Drug Administration (FDA) approved duloxetine for diabetic peripheral neuropathic pain, and recent recommendations have suggested that duloxetine may be effective for oxaliplatin-induced neuropathic pain [15]. In this study, we demonstrated that increased frequency of licking or shaking and withdrawal responses to acetone and the von Frey hair stimulations induced by oxaliplatin injection significantly decreased after the administration of duloxetine. Duloxetine showed a dose-dependent effect. The lowest dose (10 mg/kg) did not show any significant analgesic effect, whereas the medium dose of duloxetine (30 mg/kg) showed a significantly potent analgesic effect. The highest dose (60 mg/kg) had a slightly stronger effect than the medium dose; however, it was reported to reduce spontaneous locomotor activity [24,25]. Also, these three different doses of duloxetine showed a time-dependent effect. Except for 60 mg/kg of duloxetine in cold allodynia, where the effect was constant from 30 to 180 min, all other doses of duloxetine showed decreased effect at late hours of the injection. In another study conducted in L5/L6 spinal nerve-ligated pain model rats, the analgesic effect of 30 mg/kg of duloxetine was reported to be significant at 6 h, but disappeared at 24 h after the injection, showing that the effect of 30 mg/kg of duloxetine lasted less than 24 h [24].

Furthermore, by using in vivo extracellular recording method, we demonstrated that the increased WDR neuronal activities from oxaliplatin injection were decreased after the injection of duloxetine. WDR neurons receive all types of somatosensory stimuli including non-nociceptive and nociceptive inputs via the afferent A- and C-fibers. WDR cells project these signals to the higher area of the central nervous system. Therefore, the spinal WDR neuron is suitable for assessing the degree of pain, as hyperexcitability is shown in pain conditions [26,27]. In our previous study conducted on rats, we demonstrated that WDR neuronal activities in response to mechanical and cold stimulations were increased after a single injection of oxaliplatin (6 mg/kg) [17]. In the present study, we showed that 30 mg/kg of duloxetine could attenuate this hyperexcitability of spinal WDR cells, which is in accordance with our behavioral data.

In subsequent experiments, we focused on the noradrenaline mechanism of duloxetine at the spinal level. Some previously published studies have reported that increased availability of endogenous noradrenaline in the spinal cord could enhance pain relief [28]. Duloxetine, as an antidepressant drug and a noradrenaline re-uptake inhibitor, may increase the release of noradrenaline in the spinal cord, and thereby enhance the contribution of the descending noradrenergic pathway [29,30]. Our previous study also demonstrated that spinal administration of α-adrenergic agonists phenylephrine and clonidine (α_1_- and α_2_-adrenergic receptor agonist, respectively), but not isoprenaline (β-adrenergic receptor agonist), decreased the allodynia evoked by oxaliplatin injection, suggesting that the inhibitory effect of noradrenaline on spinal oxaliplatin-induced allodynia is mediated mainly by the activation of spinal α_2_ and/or α_1_-adrenergic receptors [17].

As the non-selective α-adrenergic receptor antagonist (phentolamine) completely blocked the analgesic effect of duloxetine, we further administered α_1_- or α_2_-adrenergic receptor antagonists (prazosin and idazoxan, respectively) to observe on which adrenergic receptor subtypes the duloxetine acted. Results showed that α_1_-adrenergic receptor antagonists completely blocked the effect of duloxetine, whereas α_2_-adrenergic receptor antagonists did not. Although it is hard to explain all of the details of these results, the α_1_-adrenergic receptor may have played an important role in the pain-attenuating effect of the duloxetine. The α_1_-adrenergic receptor is known to be present in presynaptic terminals of inhibitory interneurons to facilitate miniature *gamma*-Aminobutyric acid (GABA) or glycine release, and in deeper lamina to excite inhibitory neurons which enhance spontaneous large amplitudes of GABA and glycinergic responses in substantia gelatinosa neurons [31], thus decreasing pain signals. In other disease models, it was reported that the enhancement of external urethral sphincter activity evoked by duloxetine injection was blocked by α_1_- but not by α_2_-adrenergic receptor antagonist under the conditions of bladder irritation, suggesting that duloxetine acts on α_1_-adrenergic receptors [32]. In our experiments, α_2_-adrenergic receptor antagonists did not block the analgesic effect of duloxetine. However, compared to the control, the anti-nociceptive effect of duloxetine was slightly but not significantly attenuated, suggesting that the α_2_-adrenergic receptor may also have contributed to the action of duloxetine in some ways.

## 4. Materials & Methods

### 4.1. Experimental Animals

Male adult C57BL/6 mice and Sprague-Dawley rats (6–8 weeks old, Daehan Biolink, Chungbuk, Korea) were housed in cages with free access to food and water and were sustained at room temperature, 23 ± 2 °C. They were kept under specific pathogen-free conditions with air conditioning and a 12 h light/dark cycle. The animals had free access to food and water during the experiments. The study was approved on 8 May 2015 by the Kyung Hee University Animal Care and Use Committee (KHUASP(SE)-15-024).

### 4.2. Behavioral Tests Experimental Protocol

To observe the time-dependent effects of different doses of duloxetine, evaluations were conducted at timepoint zero (before the administration of duloxetine), and at 30, 60, 90, and 180 min after the injection. To measure the effects of antagonists on the analgesic effect of duloxetine, behavioral tests were performed twice; 20 min prior to the administration of antagonists and 60 min after the injection of duloxetine.

### 4.3. Allodynic Behavior Measurements

Allodynic behavior measurements were conducted by using acetone and the von Frey filament (Linton Instrumentation, Norfolk, UK). Mice were habituated to handling and to all testing procedures for a week before the initiation of the experiments. The experimenters were blinded to oxaliplatin and any other drug injections. Cold sensitivity was measured by the acetone test [33]. Mice were placed in a clear plastic box (12 × 8 × 6 cm) with a wire mesh floor and allowed to habituate for 30 min prior to the testing. Acetone (10 μL, Reagents Chemical Ltd., Gyonggi-do, Korea) was sprayed onto the plantar skin of each hind paw three times and the frequency of licking and shaking of the affected paw was counted during 30 s and averaged after the acetone had been sprayed.

Mechanical sensitivity was measured by the von Frey filament test [34]. Mice were placed in a clear plastic box (12 × 8 × 6 cm) with a wire mesh floor and allowed to habituate for 30 min before testing. A von Frey filament with a bending force of 0.4 g was applied to the mid-plantar skin (avoiding the base of the tori) of each hind paw 10 times, with each application held for 3 s [35]. The number of withdrawal responses to the von Frey filament applications from both hind paws were counted and then expressed as an overall percentage response.

### 4.4. Oxaliplatin Administration

Oxaliplatin (Sigma, St. Louis, MO, USA) was dissolved in a 5% glucose solution at a concentration of 2 mg/mL and was intraperitoneally (i.p.) injected at 6 mg/kg [7]. The control group received the same volume of 5% glucose solution (i.p.).

### 4.5. Duloxetine and α-Adrenergic Receptor Antagonists Administration

Duloxetine was dissolved in distilled water (D.W.). Different doses of duloxetine (10, 30, and 60 mg/kg) were administered (i.p.). To test which adrenergic receptor subtypes mediated the anti-allodynic effects of duloxetine in oxaliplatin-administered mice, antagonists were administered intrathecally 20 min prior to duloxetine treatments. Non-selective α-adrenergic antagonists (phentolamine, 20 μg, Sigma), α_1_-adrenergic receptor antagonists (prazosin, 10 μg, Sigma), and α_2_-adrenergic receptor antagonists (idazoxan, 10 μg, Sigma) were administered in volumes of 5 μL. The dose of each antagonist was determined based on previously conducted studies showing the selective and effective antagonistic action against adrenergic receptor-mediated responses [36,37,38].

### 4.6. In Vivo Extracellular Recording

Extracellular recordings were made from Sprague-Dawley rats, three to five days after the administration of oxaliplatin, when rats exhibited significant mechanical and cold allodynia. Extracellular recordings were carried out as previously described [17]. In brief, rats were anesthetized with urethane (Sigma; 1.5 g/kg, i.p.). The spinal cords of the animals, which were fixed in a stereotaxic frame, was exposed from T13–L2 and irrigated with oxygenated (95% O_2_-5% CO_2_ gas) Krebs solution (in mM: 117 NaCl, 3.6 KCl, 2.5 CaCl_2_, 1.2 MgCl_2_, 1.2 NaH_2_PO_4_, 11 glucose, and 25 NaHCO_3_) at a flow rate of 10–15 mL/min at 38 ± 1 °C. Based on their responses to brush, pressure, pinch, and acetone stimulations, the WDR cells were classified. Extracellular single-unit recordings were made with a low-impedance insulated tungsten microelectrode (impedance of 10 MΩ, FHC, Bowdoin, ME, USA). For mechanical stimuli, brush, press, and pinch stimulations were applied to the lateral and ventral surfaces of the hind paw. Brush stimulus was given by brushing the receptive field five times with a camel brush. Press stimulus was given by pressing the receptive field for 4 s using the blunt tip of the camel brush with a diameter of 0.5 cm and a magnitude of about 20 g. Pinch stimulation was given by pinching the skin using toothed forceps (11022-14, Fine Science Tools, Heidelberg, Germany) for 3 s. For cold stimulation, 10 μL of acetone drop was applied to the receptive fields.

### 4.7. Statistical Analysis

The data is presented as mean ± S.E.M. and was analyzed by the paired or one-way ANOVA followed by the Bonferroni multiple comparison test to determine statistical differences among the groups. *p* < 0.05 was considered as statistically significant.

## 5. Conclusions

In conclusion, our results demonstrate that a medium dose (30 mg/kg) of duloxetine can effectively attenuate oxaliplatin-induced cold and mechanical allodynia, and that the anti-allodynic effect of duloxetine is mediated by the spinal α_1_-adrenergic receptors. These findings suggest that 30 mg/kg of duloxetine can be considered as an effective drug to attenuate the allodynia induced by oxaliplatin.

## References

[1] Andre T., Boni C., Mounedji-Boudiaf L., Navarro M., Tabernero J., Hickish T., Topham C., Zaninelli M., Clingan P., Bridgewater J. (2004). Oxaliplatin, fluorouracil, and leucovorin as adjuvant treatment for colon cancer. N. Engl. J. Med..

[2] De Gramont A., Figer A., Seymour M., Homerin M., Hmissi A., Cassidy J., Boni C., Cortes-Funes H., Cervantes A., Freyer G. (2000). Leucovorin and fluorouracil with or without oxaliplatin as first-line treatment in advanced colorectal cancer. J. Clin. Oncol..

[3] Belani C.P. (2004). Recent updates in the clinical use of platinum compounds for the treatment of lung, breast, and genitourinary tumors and myeloma. Semin. Oncol..

[4] Balayssac D., Ferrier J., Pereira B., Gillet B., Petorin C., Vein J., Libert F., Eschalier A., Pezet D. (2015). Prevention of oxaliplatin-induced peripheral neuropathy by a polyamine-reduced diet-neuroxapol: Protocol of a prospective, randomised, controlled, single-blind and monocentric trial. BMJ Open.

[5] Beijers A.J., Mols F., Vreugdenhil G. (2014). A systematic review on chronic oxaliplatin-induced peripheral neuropathy and the relation with oxaliplatin administration. Support. Care Cancer.

[6] Ling B., Coudore F., Decalonne L., Eschalier A., Authier N. (2008). Comparative antiallodynic activity of morphine, pregabalin and lidocaine in a rat model of neuropathic pain produced by one oxaliplatin injection. Neuropharmacology.

[7] Ling B., Coudore-Civiale M.A., Balayssac D., Eschalier A., Coudore F., Authier N. (2007). Behavioral and immunohistological assessment of painful neuropathy induced by a single oxaliplatin injection in the rat. Toxicology.

[8] Wolf S., Barton D., Kottschade L., Grothey A., Loprinzi C. (2008). Chemotherapy-induced peripheral neuropathy: Prevention and treatment strategies. Eur. J. Cancer.

[9] Argyriou A.A., Polychronopoulos P., Iconomou G., Chroni E., Kalofonos H.P. (2008). A review on oxaliplatin-induced peripheral nerve damage. Cancer Treat. Rev..

[10] Sultan A., Gaskell H., Derry S., Moore R.A. (2008). Duloxetine for painful diabetic neuropathy and fibromyalgia pain: Systematic review of randomised trials. BMC Neurol..

[11] Chappell A.S., Ossanna M.J., Liu-Seifert H., Iyengar S., Skljarevski V., Li L.C., Bennett R.M., Collins H. (2009). Duloxetine, a centrally acting analgesic, in the treatment of patients with osteoarthritis knee pain: A 13-week, randomized, placebo-controlled trial. Pain.

[12] Skljarevski V., Desaiah D., Liu-Seifert H., Zhang Q., Chappell A.S., Detke M.J., Iyengar S., Atkinson J.H., Backonja M. (2010). Efficacy and safety of duloxetine in patients with chronic low back pain. Spine.

[13] McIntyre R.S., Panjwani Z.D., Nguyen H.T., Woldeyohannes H.O., Alsuwaidan M., Soczynska J.K., Lourenco M.T., Konarski J.Z., Kennedy S.H. (2008). The hepatic safety profile of duloxetine: A review. Expert Opin. Drug Metab. Toxicol..

[14] Smith E.M., Pang H., Cirrincione C., Fleishman S., Paskett E.D., Ahles T., Bressler L.R., Fadul C.E., Knox C., Le-Lindqwister N. (2013). Effect of duloxetine on pain, function, and quality of life among patients with chemotherapy-induced painful peripheral neuropathy: A randomized clinical trial. JAMA.

[15] Hershman D.L., Lacchetti C., Dworkin R.H., Lavoie Smith E.M., Bleeker J., Cavaletti G., Chauhan C., Gavin P., Lavino A., Lustberg M.B. (2014). Prevention and management of chemotherapy-induced peripheral neuropathy in survivors of adult cancers: American society of clinical oncology clinical practice guideline. J. Clin. Oncol..

[16] Pertovaara A. (2006). Noradrenergic pain modulation. Prog. Neurobiol..

[17] Choi S., Yamada A., Kim W., Kim S.K., Furue H. (2017). Noradrenergic inhibition of spinal hyperexcitation elicited by cutaneous cold stimuli in rats with oxaliplatin-induced allodynia: Electrophysiological and behavioral assessments. J. Physiol. Sci..

[18] Kim W., Kim M.J., Go D., Min B.-I., Na H.S., Kim S.K. (2016). Combined effects of bee venom acupuncture and morphine on oxaliplatin-induced neuropathic pain in mice. Toxins.

[19] Baker D.E. (2003). Oxaliplatin: A new drug for the treatment of metastatic carcinoma of the colon or rectum. Rev. Gastroenterol. Disord..

[20] Farquhar-Smith P. (2011). Chemotherapy-induced neuropathic pain. Curr. Opin. Support. Palliat. Care.

[21] Kannarkat G., Lasher E.E., Schiff D. (2007). Neurologic complications of chemotherapy agents. Curr. Opin. Neurol..

[22] Carrato A., Gallego J., Díaz-Rubio E. (2002). Oxaliplatin: Results in colorectal carcinoma. Crit. Rev. Oncol. Hematol..

[23] Finnerup N.B., Sindrup S.H., Jensen T.S. (2010). The evidence for pharmacological treatment of neuropathic pain. Pain.

[24] Iyengar S., Webster A.A., Hemrick-Luecke S.K., Xu J.Y., Simmons R.M.A. (2004). Efficacy of duloxetine, a potent and balanced serotonin-norepinephrine reuptake inhibitor in persistent pain models in rats. J. Pharmacol. Exp. Ther..

[25] Munro G., Storm A., Hansen M.K., Dyhr H., Marcher L., Erichsen H.K., Sheykhzade M. (2012). The combined predictive capacity of rat models of algogen-induced and neuropathic hypersensitivity to clinically used analgesics varies with nociceptive endpoint and consideration of locomotor function. Pharmacol. Biochem. Behav..

[26] Giordano J. (2005). The neurobiology of nociceptive and anti-nociceptive systems. Pain Phys..

[27] Jensen T.S., Gottrup H., Sindrup S.H., Bach F.W. (2001). The clinical picture of neuropathic pain. Eur. J. Pharmacol..

[28] Alba-Delgado C., Borges G., Sanchez-Blazquez P., Ortega J.E., Horrillo I., Mico J.A., Meana J.J., Neto F., Berrocoso E. (2012). The function of α_2_-adrenoceptors in the rat locus coeruleus is preserved in the chronic constriction injury model of neuropathic pain. Psychopharmacology.

[29] Alba-Delgado C., Mico J.A., Sanchez-Blazquez P., Berrocoso E. (2012). Analgesic antidepressants promote the responsiveness of locus coeruleus neurons to noxious stimulation: Implications for neuropathic pain. Pain.

[30] Koch S., Hemrick-Luecke S.K., Thompson L.K., Evans D.C., Threlkeld P.G., Nelson D.L., Perry K.W., Bymaster F.P. (2003). Comparison of effects of dual transporter inhibitors on monoamine transporters and extracellular levels in rats. Neuropharmacology.

[31] Baba H., Goldstein P.A., Okamoto M., Kohno T., Ataka T., Yoshimura M., Shimoji K. (2000). Norepinephrine facilitates inhibitory transmission in substantia gelatinosa of adult rat spinal cord (part 2) effects on somatodendritic sites of gabaergic neurons. Anesthesiology.

[32] Thor K.B., Katofiasc M.A. (1995). Effects of duloxetine, a combined serotonin and norepinephrine reuptake inhibitor, on central neural control of lower urinary tract function in the chloralose-anesthetized female cat. J. Pharmacol. Exp. Ther..

[33] Flatters S.J., Bennett G.J. (2004). Ethosuximide reverses paclitaxel- and vincristine-induced painful peripheral neuropathy. Pain.

[34] Joseph E.K., Levine J.D. (2009). Comparison of oxaliplatin- and cisplatin-induced painful peripheral neuropathy in the rat. J. Pain.

[35] Shibata K., Sugawara T., Fujishita K., Shinozaki Y., Matsukawa T., Suzuki T., Koizumi S. (2011). The astrocyte-targeted therapy by bushi for the neuropathic pain in mice. PLoS ONE.

[36] Johnson J.D., Campisi J., Sharkey C.M., Kennedy S.L., Nickerson M., Greenwood B.N., Fleshner M. (2005). Catecholamines mediate stress-induced increases in peripheral and central inflammatory cytokines. Neuroscience.

[37] Nelson L.E., Lu J., Guo T., Saper C.B., Franks N.P., Maze M. (2003). The α_2_-adrenoceptor agonist dexmedetomidine converges on an endogenous sleep-promoting pathway to exert its sedative effects. Anesthesiology.

[38] Zarrindast M.R., Homayoun H., Khavandgar S., Fayaz-Dastgerdi M., Fayaz-Dastgerdi M. (2002). The effects of simultaneous administration of α_2_-adrenergic agents with l-name or l-arginine on the development and expression of morphine dependence in mice. Behav. Pharmacol..

